# Accuracy of haplotype estimation in a region of low linkage disequilibrium

**DOI:** 10.1186/1471-2156-6-S1-S80

**Published:** 2005-12-30

**Authors:** Christy L Avery, Lisa J Martin, Jeff T Williams, Kari E North

**Affiliations:** 1 Department of Epidemiology, University of North Carolina, Chapel Hill, North Carolina, USA; 2 Center for Epidemiology and Biostatistics, Cincinnati Children's Hospital Medical Center, Cincinnati, Ohio, USA; 3 Department of Genetics, Southwest Foundation for Biomedical Research, San Antonio, Texas, USA; 4 Southwest National Primate Research Center, Southwest Foundation for Biomedical Research, San Antonio, Texas, USA

## Abstract

We compared the accuracy of haplotype inferences at a 6 Mb region on chromosome 7 where significant linkage between a brain oscillation phenotype and a cholinergic muscarinic receptor gene was previously reported. Individual haplotype assignments and haplotype frequencies were estimated using 5, 10, and 14 consecutive Illumina single-nucleotide polymorphisms (SNPs) within the 1-LOD unit support interval of the chromosome 7 linkage peak. Initially, haplotypes were constructed incorporating phase information provided by relatives using the pedigree analysis package MERLIN. Population-based haplotypes were inferred using the haplotype estimation software HAPLO.STATS and PHASE, using unrelated individuals.

The 14 SNPs within this region exhibited markedly low linkage disequilibrium, and the average D' estimate between SNPs was 0.18 (range: 0.01–0.97). In comparison to the family-based haplotypes calculated in MERLIN, the computational inferences of individual haplotype assignments were most accurate when considering 5 consecutive SNPs, but decayed dramatically when considering 10 or 14 SNPs in both PHASE and HAPLO.STATS. When comparing the two haplotype inference methods, both PHASE and HAPLO.STATS performed poorly. These analyses underscore the difficulties of haplotype estimation in the presence of low linkage disequilibrium and stress the importance of careful consideration of confidence measures when using estimated haplotype frequencies and individual assignments in biomedical research.

## Background

The advent of inexpensive high-throughput single-nucleotide polymorphism (SNP) genotyping [1,2] and very recent bioinformatic and statistical advances [3] now facilitate genome-wide SNP association analyses in large samples of individuals. Risch and Merikangas [2] argue that association analyses are more powerful for the detection of common variants that affect common disease. Others note that it is easier to recruit unrelated individuals than to collect the large numbers of pedigrees required for successful linkage studies [4]. However, very high marker densities are required for whole-genome association studies in large outbred populations, with estimates ranging from 200,000 to one million markers needed to achieve a reasonable likelihood of detecting an association [1,5].

The International HapMap Project [5] was initiated to define haplotype patterns across the genome, with the goal of developing a map of non-redundant tagSNPs. TagSNPs allow the identification of unique haplotypes while genotyping a fewer number of total SNPs for association analyses. However, the true density of the marker map needed is debated, with recent studies suggesting a more complex haplotype architecture of genes across the human genome than was previously suggested [6].

In this study we assessed the accuracy of computational inferences of individual haplotypes and haplotype frequencies at a region on chromosome 7 where Jones et al. [7] detected significant linkage to a target case frontal theta band visual evoked brain oscillation phenotype in Collaborative Study of the Genetics of Alcoholism (COGA) participants. Haplotypes were estimated using pedigree information in MERLIN and compared to population-based haplotypes using several combinations of the 14 SNPs identified under the 1-LOD unit support interval and haplotype estimation algorithms in PHASE and HAPLO.STATS.

## Methods

COGA began in 1989 to elucidate genetic mechanisms that influence susceptibility to alcohol abuse, alcohol dependence, and related phenotypes [8]. The COGA dataset provided for Genetic Analysis Workshop 14 (GAW14) includes 1,026 non-Hispanic White family members from 91 pedigrees (ranging in size 5–32 individuals) collected from 6 United States sites. Additionally, individuals were genotyped for 4,763 Illumina SNPs spread across the genome.

### SNP selection

We selected the 14 SNPS from the cleaned Illumina SNP dataset (rs1464798, rs880290, rs1476640, rs1859646, rs768055, rs17229, rs940864, rs727714, rs969356, rs1860482, rs2056553, rs700273, rs802200, rs850545) that were within the 1-LOD unit support interval (6 Mb) for the quantitative trait locus (QTL) on chromosome 7 initially reported by Jones et al. [7]. Three groupings of SNPs were initially examined; the first 5 SNPs (rs1464798, rs880290, rs1476640, rs768055, rs1859646), the first 10 SNPs (rs1464798, rs880290, rs1476640, rs768055, rs1859646, rs17229, rs940864, rs727714, rs969356, rs2056553) and all 14 SNPs. This grouping of 5, 10, and 14 SNPs was chosen to examine the impact of the number of SNPs on haplotype estimation, while roughly dividing the sample in thirds.

### Statistical methods

Individual haplotypes were first determined using all related individuals with the pedigree analysis package MERLIN and the -best option, which provides haplotypes corresponding to the most likely pattern of gene flow within a pedigree [9]. Four families (*n *= 21 individuals) also had members removed to comply with MERLIN pedigree size restrictions.

To examine population level statistics, we selected 1 individual with complete SNP data for the 14 SNPs from each of the 91 families. We determined both the amount of linkage disequilibrium (LD) between SNPs and Hardy-Weinberg equilibrium using the computer program HAPLOVIEW [10]. We used PHASE [11] and HAPLO.STATS [12] to generate population-based haplotypes, using unrelated individuals. PHASE employs a Bayesian method of haplotype reconstruction and uses Gibbs sampling to obtain an approximate sample from the posterior distribution of Pr(haplotype|genotype) [13]. HAPLO.STATS uses the expectation-maximization algorithm and progressively inserts batches of loci into haplotypes.

### Haplotype accuracy measures

Excoffier and Slatkin [14] proposed *I*_*F *_and *I*_*H*_, where *I*_*F *_is a metric of agreement between family-based and population-based haplotypes and is given by . *p*_*ek *_and *p*_*tk *_represent the population-based and family-based frequencies for the *k*^th ^haplotype and *h *is the number of possible haplotypes. *I*_*H *_compares the number of population-based haplotypes to the number of actual haplotypes and ranges from 0 to 1 (total correspondence between estimated and family-based haplotypes) and is defined as *I*_*H *_= 2(*m*_*true *_- *m*_*missed*_) / *m*_*true *_+ *m*_*est*_, where *m*_*true *_represents the number of actual haplotypes, *m*_*est *_is the number of population-based haplotypes, and *m*_*missed *_corresponds to the number of family-based haplotypes that were not inferred. To assess individual haplotype inference we also calculated an overall error rate, defined as the proportion of individuals whose population-based haplotype differed from the true haplotype.

## Results

**Figure 1:**
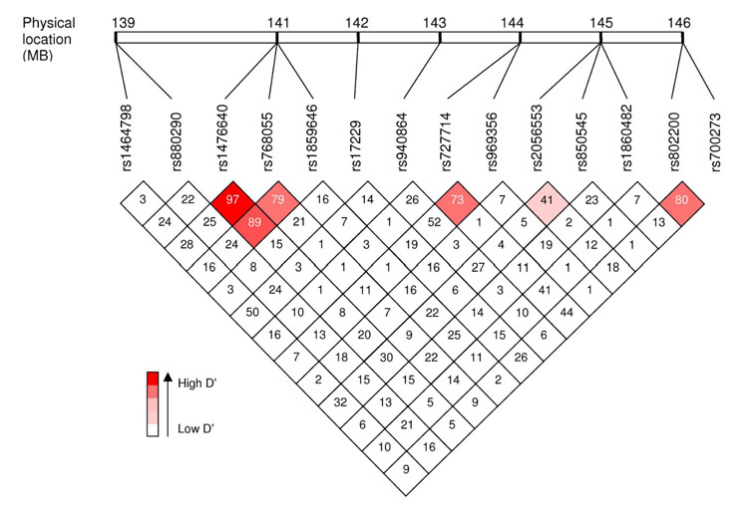
LD structure of 14 Illumina SNPs within the 1-LOD unit drop support interval for the QTL on chromosome 7 initially reported by Jones et al. [7].

All 14 SNPs were in Hardy-Weinberg equilibrium, with minor allele frequencies ranging from 0.269–0.473. The SNPs exhibited markedly low LD, as the average D' (Figure 1) and R^2 ^estimates between the SNPs were 0.18 (range: 0.01–0.97) and 0.04 (range: 0–0.64), respectively. Eleven individuals (12%) had haplotypes with at least 1 SNP for which phase could not be determined using MERLIN and were excluded from all subsequent analyses.

Table 1 reports the distribution of haplotype and the respective frequencies, as calculated in MERLIN using the 5, 10, and 14 SNPs. Four haplotypes had frequencies greater than 10%, 12 had frequencies greater than 1%, and 2 had frequencies less than 1%.

### Accuracy of haplotype estimation

The accuracy of haplotype estimation when incorporating 5 consecutive SNPs was assessed by comparing true haplotype frequencies calculated in MERLIN against population-based haplotype frequencies estimated by PHASE and HAPLO.STATS (Table 1). Although estimated haplotype frequencies exhibited moderate levels of accuracy for haplotypes with high frequencies, both programs missed rare haplotypes and specified incorrect haplotypes.

We also quantified the accuracy of the haplotype frequencies and the agreement between individual family-based and population-based haplotype estimates across the 5-SNP, 10-SNP, and 14-SNP haplotypes (Table 2). When making haplotype inferences using 5 consecutive SNPs (average D' = 0.408), both PHASE and HAPLO.STATS performed similarly, with overall error rates of 0.275 and 0.287, respectively. To determine the importance of the underlying LD structure, we also chose 5 SNPs (rs1464798, rs880290, rs17229, rs2056553, rs1860482) with generally low D' values (average D' = 0.085). The overall error rate increased to 0.56 when inferring haplotypes in PHASE using the SNP set with lower D' values.

When the number of SNPs analyzed was increased to 10 and 14, PHASE appeared to perform slightly better than HAPLO.STATS, as indicated by higher *I*_*H *_and *I*_*F *_estimates. However, with this number of SNPs both programs estimated haplotypes with substantial inaccuracy.

We also were interested to determine if the inclusion of additional SNPs influenced haplotype inference of a subset. Thus, the 10- and 14-SNP haplotypes were truncated at the fifth SNPs and accuracy was assessed (Table 2). PHASE generally outperformed HAPLO.STATS. Of interest, the 5-SNP haplotype demonstrating the lowest overall error rate was observed for haplotypes in the 10-SNP set truncated at the fifth SNP for both PHASE and HAPLO.STATS. This may reflect the fact that the additional SNPs are in some degree of LD with the first 5 SNPs. However, the reduction in the overall error rate appears to be ultimately offset as SNPs that are further away are incorporated.

## Discussion

In this paper, we compared family-based and population-based individual haplotype estimates over a 6 Mb region corresponding to the linkage signal previously reported by Jones et al. [7]. Individual haplotype inferences calculated in PHASE and HAPLO.STATS were most accurate when considering 5 consecutive SNPs, but decayed dramatically when evaluating 10 or 14 SNPs. These findings are concordant with those of Xu et al. [15] and Adkins et al. [16], who demonstrated that the accuracy of computational haplotype inference improves as the magnitude of LD among sites increases. However, our data demonstrate high levels of inaccuracy, most likely reflecting the low LD structure of the region examined.

When comparing the two haplotype inference methods, both PHASE and HAPLO.STATS performed similarly, although PHASE slightly outperformed HAPLO.STATS. These findings are in agreement with previous studies comparing various methods of haplotype assignment and haplotype frequency estimation, which have consistently shown similar levels of accuracy and consistency across software packages and computational methods [15-18]. However, our study is the first to evaluate HAPLO.STATS.

Although the decay of efficiency in haplotype estimation is most likely due to the increasing number of possible haplotypes, these results are important considering the availability of 100,000 SNP panels (both from Affymetrix and Illumina). Thus, more investigators will face the challenge of creating haplotypes from large SNP sets. Our results suggest that haplotypes estimated from population-based data should be interpreted with caution. Even though many features of haplotype inference are found to be consistent from one dataset to the next, it is not yet clear how general these tendencies will prove to be in the context of very low LD (e.g., how robust to variation in LD structure from one dataset to another, or what size SNP blocks appears optimal), and future research is warranted.

While both programs had high levels of inaccuracy, statistical measures of confidence, such as a posterior probability estimate for each individual haplotype, are provided. For example, in the 5-SNP haplotypes estimated in PHASE, the incorrectly specified haplotypes had a mean posterior probability estimate of 0.52 (range: 0.34–0.66). Clearly, such uncertainty in haplotype assignment should be incorporated into subsequent statistical analyses incorporating these haplotypes. Unfortunately, such practices do not routinely appear in the literature.

## Conclusion

Both haplotype estimation packages performed similarly and poorly when 5, 10, and 14 SNP sets were considered, although PHASE slightly outperformed HAPLO.STATS. Thus, our findings underscore the difficulties of computational haplotype inference under less-than-ideal conditions (linkage region with low LD) and stress the importance of careful consideration of confidence measures when employing estimated haplotypes in biomedical research. Further, the definition of haplotype blocks should be considered carefully on a case-by-case basis, with careful attention to the number of underlying sites and the pattern of LD.

## Abbreviations

COGA: Collaborative Study of the Genetics of Alcoholism

GAW14: Genetic Analysis Workshop 14

LD: Linkage disequilibrium

QTL: Quantitative trait locus

SNP: Single-nucleotide polymorphism

## Authors' contributions

CLA and LJM performed statistical analyses, interpreted results, and collaborated to write the manuscript; JTW and KEN aided in interpretation of results and collaborated to write the manuscript. All authors read and approved the final manuscript.

## Figures and Tables

**Table 1:** Frequency of population-based and family-based haplotypes using 14 Illumina SNPs with low LD

	5-SNP Haplotype Frequencies		
	
Haplotype	MERLIN	PHASE	HAPLO.STATS
22212	0.2188	0.225	0.2375
12212	0.1375	0.1438	0.1438
22121	0.125	0.0688	0.075
12121	0.1125	0.1563	0.1625
11121	0.0813	0.0938	0.0875
21121	0.0688	0.0688	0.0625
21212	0.05	0.075	0.0625
11212	0.0438	0.0063	0.0063
22222	0.0438	0.0438	0.0438
11222	0.0188	0.0313	0.0313
12222	0.0188	-	-
21211	0.0188	-	0.0188
22211	0.0188	0.0438	0.025
12122	0.0125	-	-
12211	0.0125	-	-
22221	0.0125	0.0188	0.0188
12221	0.0063	-	-
22111	0.0063	0.0063	-
22122	-^a^	0.0125	0.0125
11221	-	0.0063	0.0063
21111	-	-	0.0063

^a ^-, not applicable.

**Table 2:** Accuracy of population-based haplotype estimates in 5-SNP, 10-SNP, and 14-SNP haplotypes.

# SNPs incorporated	Algorithm	*I*_*H*_	*I*_*F*_	Overall error rate
5 SNPs	PHASE	0.788	0.837	0.275
	HAPLO.STATS	0.765	0.850	0.287
10 SNPs	PHASE	0.525	0.465	0.687
	HAPLO.STATS	0.361	0.293	0.855
14 SNPs	PHASE	0.225	0.189	0.900
	HAPLO.STATS	0.136	0.092	0.950
Truncated at 5^th ^SNP				
10 SNPs	PHASE	0.882	0.856	0.200
	HAPLO.STATS	0.811	0.881	0.262
14 SNPs	PHASE	0.857	0.875	0.237
	HAPLO.STATS	0.703	0.850	0.337

## References

[B1] Kruglyak L (1999). Prospects for whole-genome linkage disequilibrium mapping of common disease genes. Nat Genet.

[B2] Risch N, Merikangas K (1996). The future of genetic studies of complex human diseases. Science.

[B3] Zondervan KT, Cardon LR (2004). The complex interplay among factors that influence allelic association. Nat Rev Genet.

[B4] Botstein D, Risch N (2003). Discovering genotypes underlying human phenotypes: past successes for mendelian disease, future approaches for complex disease. Nat Genet.

[B5] Gibbs RA, Belmont JW, Hardenbol P, Willis TD, Yu FL, Yang HM, Ch'ang LY, Huang W, Liu B, Shen Y, Tam PKH, Tsui LC, Waye MMY, Wong JTF, Zeng CQ, Zhang QR, Chee MS, Galver LM, Kruglyak S, Murray SS, Oliphant AR, Montpetit A, Hudson TJ, Chagnon F, Ferretti V, Leboeuf M, Phillips MS, Verner A, Kwok PY, Duan SH, Lind DL, Miller RD, Rice JP, Saccone NL, Taillon-Miller P, Xiao M, Nakamura Y, Sekine A, Sorimachi K, Tanaka T, Tanaka Y, Tsunoda T, Yoshino E, Bentley DR, Deloukas P, Hunt S, Powell D, Altshuler D, Gabriel SB, Qiu RZ, Ken A, Dunston GM, Kato K, Niikawa N, Knoppers BM, Foster MW, Clayton EW, Wang VO, Watkin J, Gibbs RA, Belmont JW, Sodergren E, Weinstock GM, Wilson RK, Fulton LL, Rogers J, Birren BW, Han H, Wang HG, Godbout M, Wallenburg JC, L'Archeveque P, Bellemare G, Todani K, Fujita T, Tanaka S, Holden AL, Lai EH, Collins FS, Brooks LD, McEwen JE, Guyer MS, Jordan E, Peterson JL, Spiegel J, Sung LM, Zacharia LF, Kennedy K, Dunn MG, Seabrook R, Shillito M, Skene B, Stewart JG, Valle DL, Clayton EW, Jorde LB, Belmont JW, Chakravarti A, Cho MK, Duster T, Foster MW, Jasperse M, Knoppers BM, Kwok PY, Licinio J, Long JC, Marshall PA, Ossorio PN, Wang VO, Rotimi CN, Royal CDM, Spallone P, Terry SF, Lander ES, Lai EH, Nickerson DA, Abecasis GR, Altshuler D, Bentley DR, Boehnke M, Cardon LR, Daly MJ, Deloukas P, Douglas JA, Gabriel SB, Hudson RR, Hudson TJ, Kruglyak L, Kwok PY, Nakamura Y, Nussbaum RL, Royal CDM, Schaffner SF, Sherry ST, Stein LD, Tanaka T (2003). The International HapMap Project. Nature.

[B6] Crawford DC, Carlson CS, Rieder MJ, Carrington DP, Yi Q, Smith JD, Eberle MA, Kruglyak L, Nickerson DA (2004). Haplotype diversity across 100 candidate genes for inflammation, lipid metabolism, and blood pressure regulation in two populations. Am J Hum Genet.

[B7] Jones KA, Porjesz B, Almasy L, Bierut L, Goate A, Wang JC, Dick DM, Hinrichs A, Kwon J, Rice JP, Rohrbaugh J, Stock H, Wu W, Bauer LO, Chorlian DB, Crowe RR, Edenberg HJ, Foroud T, Hesselbrock V, Kuperman S, Nurnberger J, O'Connor SJ, Schuckit MA, Stimus AT, Tischfield JA, Reich T, Begleiter H (2004). Linkage and linkage disequilibrium of evoked EEG oscillations with CHRM2 receptor gene polymorphisms: implications for human brain dynamics and cognition. Int J Psychophysiol.

[B8] Reich T (1996). A genomic survey of alcohol dependence and related phenotypes: results from the Collaborative Study on the Genetics of Alcoholism (COGA). Alcohol Clin Exp Res.

[B9] Abecasis GR, Cherny SS, Cookson WO, Cardon LR (2002). Merlin – rapid analysis of dense genetic maps using sparse gene flow trees. Nat Genet.

[B10] Haploview. http://www.broad.mit.edu/personal/jcbarret/haplo/.

[B11] PHASE. http://www.stat.washington.edu/stephens/software.html.

[B12] HAPLO.STATS. http://mayoresearch.mayo.edu/mayo/research/biostat/.

[B13] Graham RR, Langefeld CD, Gaffney PM, Ortmann WA, Selby SA, Baechler EC, Shark KB, Ockenden TC, Rohlf KE, Moser KL, Brown WM, Gabriel SE, Messner RP, King RA, Horak P, Elder JT, Stuart PE, Rich SS, Behrens TW (2001). Genetic linkage and transmission disequilibrium of marker haplotypes at chromosome 1q41 in human systemic lupus erythematosus. Arthritis Res.

[B14] Excoffier L, Slatkin M (1995). Maximum-likelihood estimation of molecular haplotype frequencies in a diploid population. Mol Biol Evol.

[B15] Xu CF, Lewis K, Cantone KL, Khan P, Donnelly C, White N, Crocker N, Boyd PR, Zaykin DV, Purvis IJ (2002). Effectiveness of computational methods in haplotype prediction. Hum Genet.

[B16] Adkins RM (2004). Comparison of the accuracy of methods of computational haplotype inference using a large empirical dataset. BMC Genet.

[B17] Zhang S, Pakstis AJ, Kidd KK, Zhao H (2001). Comparisons of two methods for haplotype reconstruction and haplotype frequency estimation from population data. Am J Hum Genet.

[B18] Fallin D, Schork NJ (2000). Accuracy of haplotype frequency estimation for biallelic loci, via the expectation-maximization algorithm for unphased diploid genotype data. Am J Hum Genet.

